# COVID-19 Vaccination Program for Undocumented Migrants: Notes from the Field of a Regional Center of General Medicine and Public Health, Canton of Vaud, Switzerland

**DOI:** 10.1007/s10903-023-01479-0

**Published:** 2023-04-20

**Authors:** Kevin Morisod, Justin Nikles, Alix Miauton, Laurence Bouche Maussang, Brigitte Pahud Vermeulen, Patrick Bodenmann

**Affiliations:** 1 Department of Vulnerabilities and Social Medicine, Center for Primary Care and Public Health (Unisanté), Lausanne, Switzerland; 2 Chair of medicine for vulnerable populations, University of Lausanne, Lausanne, Switzerland; 3 Department of Ambulatory Care, Center for Primary Care and Public Health (Unisanté), Lausanne, Switzerland; 4 Center for Primary Care and Public Health (Unisanté), Lausanne, Switzerland

**Keywords:** Undocumented migrants, COVID-19 vaccination, Health equity

## Abstract

The COVID-19 pandemic highlighted health inequities for vulnerable populations and the need for more equitable care and access to vaccination. This article described the implementation of a COVID-19 vaccination program for undocumented migrants in a regional academic center of general medicine and public health (Unisanté). The vaccination program’s specific components included: triple coordination between the health authorities, the regional center and community partners, a walk-in and free service, no health insurance required, qualified nursing and administrative staff with previous experience with vulnerable populations, translated information materials and interpreters, a guarantee of confidentiality and a widespread communication campaign within the communities. In total, 2’351 undocumented migrants from 97 nationalities received at least one dose of mRNA COVID-19 vaccine (Spikevax) and 2242 were considered fully vaccinated. Although it was hard to assess its global effectiveness, the program vaccinated a significant number of undocumented adult migrants in the Canton of Vaud. The difficulties linked to the pandemic context, the heavy workload for healthcare staff and the limited resources were overcome by strong collaborations between the different actors involved throughout the program. Targeted public health policies, such as vaccination programs for undocumented migrants, are essential to guarantee equitable care, especially in pandemic times.

## Background

The COVID-19 pandemic widened the health inequity gaps in vulnerable populations. In high-income countries, studies have consistently highlighted the highest burden of the pandemic on the most socio-economically deprived residents [1–3]. In October 2021, the WHO published a report entitled *“COVID-19 and the social determinants of health and health equity”*, which summarized the most crucial health equity issues during this pandemic and highlighted the high burden of the pandemic on migrant populations [4]. The report noted the risk of additional exposure to the Sars-CoV-2 virus due to occupational and living conditions and less access to protective measures. It advocated for more equitable access to COVID-19 vaccination for these populations [4].

Among them, undocumented migrants – people living in a country without legal documents and permits^1^ – are particularly at risk of health inequities. Indeed, their living and social conditions (high population density, belonging to a minority ethnic group, cultural and language barriers, social deprivation, and being “essential workers”) increase their exposure to COVID-19 [2, 5–8]. Moreover, they have limited healthcare access, especially if they are uninsured. In this context, ensuring equitable access to vaccination for undocumented migrants is not only an ethical priority but also a significant public health issue to prevent new outbreaks [8–11]. A recent review highlighted the numerous barriers faced by undocumented migrants to access COVID-19 vaccination, including systemic restrictions and practical barriers both on the supply- and demand-side [8]. For example, the authors described the lack of interpreters in vaccination centers and the lack of adaptation of the vaccination campaigns to the living and working conditions of undocumented migrants as significant practical supply-side barriers to COVID-19 vaccination. Moreover, the undocumented migrants’ mistrust of the government and the fear of transfer of personal data to immigration authorities, associated with a low perception of the threat of COVID-19 and the large circulation of fake news regarding vaccination were all identified as critical demand-side barriers to COVID-19 vaccination [8].

Although complex, the challenge of ensuring equitable access to vaccination for undocumented migrants is achievable. Through this article, we aimed to describe a vaccination program for undocumented and uninsured migrants conducted at Unisanté, a regional center of general medicine and public health located in the Canton of Vaud, Switzerland’s third most populous canton with estimated 800’000 residents in 2021, or 10% of the total population of the country.

## Vaccination Program

The three main steps allowing the vaccination of undocumented migrants were: the communication campaign, the implementation of the vaccination program and the vaccination uptake.

### Communication Campaign

Communication about the vaccination program was essential due to the lack of access to public health information and the poorer health literacy of undocumented migrants. The communication campaign was explicitly conducted in communities and adapted to be readable, understandable, and generate action. Many documents have been translated due to the low French proficiency of a significant part of this population. Moreover, the communication campaign was explicitly delivered in the communities. We contacted at least 50 community partners (such as migrant associations, churches, NGOs, etc.). They were informed about the vaccination program and played a crucial role in promoting it.

First, the community partners shared the communication campaign messages informing of the COVID-19 vaccination message through existing communities’’ online social network groups, such as WhatsApp or Telegram. For example, a physician formerly working at Unisanté and his wife, members of the Asian community in Switzerland, sent a message translated into both Cantonese and Mandarin to a community’s WeChat group with thousands of Asian members, including many undocumented migrants. Second, the communication campaign promoted the vaccination program through influencers such as community-associated media (e.g. AlbInfo, an Albanian-speaking TV channel). Third, the communication campaign distributed documents inside the communities informing about the COVID-19 vaccination (type of vaccines, side effects, etc.) and translated them into ten languages.

### Implementation of The Vaccination Program

Cantonal health authorities mandated Unisanté in May 2021 to initiate a vaccination program for uninsured undocumented migrants. At that time, COVID-19 vaccination was already available for the general population in vaccination centers, general practitioners’ offices and hospitals. However, the requirement to show a health insurance card made these places inaccessible for uninsured undocumented migrants.

A multidisciplinary working group composed of administrative, medical, nursing and pharmacy managers, with expertise in vaccination and migrant population, was formed to implement the program. The system already in place - intended for the general population - was adapted to address the barriers to healthcare that undocumented migrants typically face. Emphasis was placed on reducing administrative barriers, ensuring confidentiality, and providing linguistically and culturally appropriate care. Specific actions taken to ensure the implementation of the program included: [1] no health insurance required for registration, [2] no appointment needed to receive the free vaccine, [3] possibility of anonymous vaccination, [4] extension of the opening hours to Saturday morning, [5] adapted administrative form to limit the collection of personal information and maintaining trust, [6] qualified nursing and administrative staff with previous experience with vulnerable populations [7] translated information materials and interpreters if needed. Since no appointments were scheduled, the organization was adapted to offer greater flexibility and to cope with the irregular patient flow. Administrative, pharmacy and nurse’s back-up was available when needed. Twice-daily checks were set up between the nurses and the pharmacy to prepare the required number of vaccine vials while avoiding wasting doses. The working group met weekly to monitor the project and perform any necessary modifications.

### Vaccination Uptake

The vaccination program began on 26 May 2021. Initially, vaccination service was provided everyday from Monday to Saturday. Three months later, vaccination was eventually suspended on Saturday owing to limited resources and low turn-out of patients.

The vaccination uptake involves the following sequence:

1. Arrival at the regional center and orientation by the administrative staff.
2. Registration for vaccination by administrative staff.
3. Waiting room before vaccination.
4. Vaccination. Before vaccination, healthcare staff checked medical history to rule out any contraindications. If participants did not speak the same language as the healthcare staff, they received a written questionnaire translated into ten languages. In many cases, participants came spontaneously with an interpreter.
5. Observation of participants. As specified by the national COVID-19 vaccination guidelines, the participants were monitored 15 min after the first vaccine dose and 5 min after the second dose to assess the occurrence of side effects.
6. Appointment for the second dose and Covid-19 vaccination certificate. Administrative staff scheduled the appointment for a second dose if needed and delivered the vaccination certificate on the way out.

## Ethics Approval

The vaccination campaign strictly followed policies on vaccination in Switzerland. After clarification of responsibility, this project (Reg-2021-01493) was approved by the Ethical Commission of Canton of Vaud (CER-VD).

## Metrics

We used administrative data of participants to monitor the COVID-19 vaccination program. Vaccination data on undocumented migrants were recorded throughout a five-month period ranging from late May through late October. The recorded data included the following variables: age (in year), nationality, health insurance status (yes or no) and appointment for the second dose (yes or no).

During the 5-month vaccination program organized by Unisanté (from 26 May to 25 October 2021), 2351 undocumented migrants without health insurance received at least one dose of mRNA COVID-19 vaccine (Spikevax). Among them, 2164 (92%) received an appointment for a second dose, as some of the participants had a history of COVID-19 and were therefore considered fully vaccinated after one dose. About 95% came back to receive it. Thus, 2055 undocumented migrants received two doses during the program, and 2242 were considered fully vaccinated. The mean participants’ age was 38 years, and 48% were female. Migrants from 97 different nationalities took part in this vaccination campaign.

## Discussion

More than 2,000 undocumented migrants without health insurance and originating from 97 different countries were fully vaccinated during the vaccination program. The main reasons for this success were the coordination between the health authorities, the regional medical center and the communities and the long-term upstream work to build and maintain trust with these populations. On one side, the top-down approach through the public health authority’s impulse gave resources to implement the vaccination program and coordinate the communication campaign. On the other side, the bottom-up approach through interventions by community partners enabled targeting those populations and maintaining trust. The example of the physician member of the Asian community in Switzerland highlighted the importance to use community social networks to promote the vaccination program. Since many members only use Asian social networks and search engines, public health messages on western social media would not reach them. Word-of-mouth within communities may also have played a role: some communities were overrepresented at times and then much less present. After being vaccinated, participants may have encouraged some of their acquaintances to come and get vaccinated. In addition, the reputation of Unisanté as social medicine center and its experience in care for vulnerable populations facilitated the implementation and maintained trust through the program. The vast majority of participants showed up for the second dose, and only a few wanted the anonymous vaccination, suggesting that an atmosphere of trust was made possible. The positive feedback of participants from countries where vaccine hesitancy is widespread also demonstrated the strength of this vaccination program. Thus, such a vaccination program is feasible and effective under the right conditions. It requires a limited increase in resources, mainly the reorganization of qualified administrative and nursing staff.

However, it was difficult to establish the global effectiveness of the vaccination program due to the lack of data regarding undocumented migrants in the Canton of Vaud. The only available study estimated in 2015 that about 12,000 undocumented migrants lived in the Canton of Vaud [12]. However, this estimate includes children (13% of the undocumented migrants) who were not eligible for COVID-19 vaccination. Then, the study did not specify the proportion of undocumented migrants having health insurance. Therefore, we cannot precisely calculate the proportion of undocumented (and uninsured) migrants vaccinated through the vaccination program. Interestingly, however, the gender ratio of participants was close to the gender ratio of this study (48.7% of female vs. 51%). Together with the vast number of nationalities, we thus estimate that we were able to vaccinate a large proportion of undocumented and uninsured migrants.

### Limitations of the Program

The vaccination program was mainly challenging due to logistical barriers, the short delay in achieving it and the heavy workload due to the COVID-19 crisis.

Multidisciplinary collaboration with stakeholders from different institutions was challenging but necessary. It helped overcome barriers like the short deadline to implement the vaccination program and coordinate the communication campaign.

An important issue was to provide quality care while being flexible about the number of people who can be vaccinated daily. Sometimes, the vaccination area was surprisingly overcrowded, as it was impossible to anticipate the number of patients a day. Especially at the beginning, there were so many people that the queue stretched out until the street and administrative staff was deployed outside to direct patients. Accordingly, administrative managers defined different scenarios and appropriate action plans. For instance, lack of space was a problem. The different rooms for the vaccination were too small during high attendance days. Therefore, some waiting rooms had to be improvised in less suitable places, and participants were invited to come back later.

Lastly, the healthcare staff was already exhausted by the Covid-19 pandemic workload [13]. Managers had to consider this and reduce working time spent on other consultations. They also organized weekly meetings with healthcare staff to discuss what could be improved in managing the vaccination program.

## Conclusion

Implementing specific COVID-19 vaccination programs targeting undocumented migrants is a practical health policy that improves equitable care and protects the entire population by reducing the risk of COVID-19 outbreaks. We hope this article will serve as evidence for supporting COVID-19 vaccination programs for undocumented migrants elsewhere.

## Footnotes

1 https://apps.who.int/iris/bitstream/handle/10665/326342/9789289051118-eng.pdf?sequence=1&isAllowed=y.
